# Specialists are central to patient chronic condition care: Medicare ACOs must adapt to this reality

**DOI:** 10.1093/haschl/qxaf228

**Published:** 2025-11-21

**Authors:** Kenton J Johnston, Alexander O Everhart, Peter F Lyu, Jason M Hockenberry

**Affiliations:** Division of General Medicine and Geriatrics, John T. Milliken Department of Medicine, Washington University School of Medicine in St. Louis, St. Louis, MO 63110, United States; Division of General Medicine and Geriatrics, John T. Milliken Department of Medicine, Washington University School of Medicine in St. Louis, St. Louis, MO 63110, United States; Center for Health Care Financing & Payment, RTI International, Research Triangle Park, NC 27709, United States; Department of Health Policy and Management, Yale School of Public Health, New Haven, CT 06510, United States

**Keywords:** accountable care organizations, alternative payment models, Medicare, chronic disease, subspecialty care

## Abstract

The accountable care organization (ACO) model centers around primary care providers (PCPs) and undervalues the central role that specialists play for many beneficiaries with chronic conditions. This assumption informs beneficiary attribution methods for Medicare ACOs, which prioritize assignment of cost and quality accountability to PCPs over specialists. Yet, in 2023, many traditional Medicare beneficiaries with chronic conditions did not have a PCP as their predominant provider of care, limiting ACOs’ ability to engage many beneficiaries with specialists as their predominant providers of care. To better engage specialists delivering chronic condition care, we recommend updating ACO policies to assign greater accountability for beneficiaries with chronic conditions to specialists.

Chronic conditions are a key driver of Medicare spending and poor health outcomes.^1^ In 2023, 52.8% of traditional Medicare (TM) beneficiaries had diagnosed asthma, chronic obstructive pulmonary disease (COPD), diabetes, heart failure, ischemic heart disease, or chronic kidney disease and accounted for 79.2% of TM spending (Figure 1). The Centers for Medicare and Medicaid Services (CMS) have prioritized moving more TM beneficiaries into accountable care relationships because policymakers believe that accountable care organizations (ACOs) can improve care and bend the cost curve for chronic conditions.^2^

**Figure 1.**
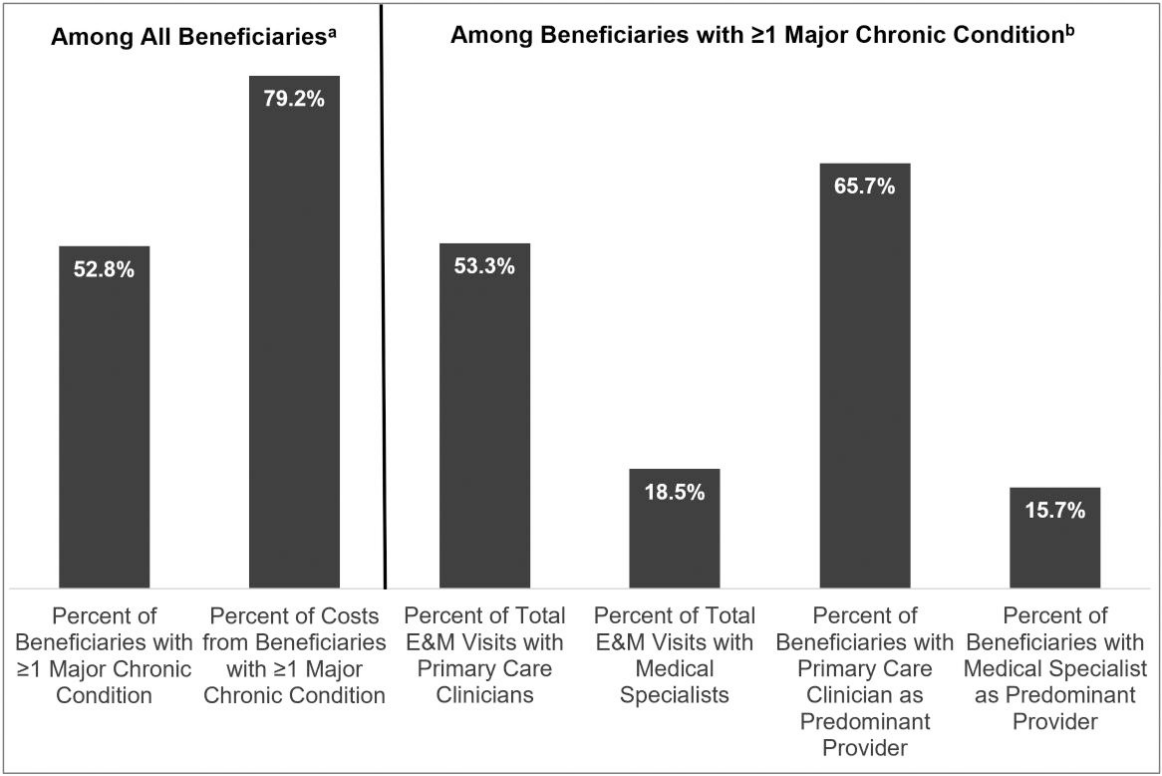
Traditional Medicare beneficiaries, 2023. ^a^Traditional Medicare beneficiaries with exclusive and continuous Part A and B enrollment in 2023 for 12 months or up until death and residing in the 50 states or Washington, DC (*n* = 27 181 937). Major chronic condition is ≥1 CCW flag in 2023 for asthma, chronic obstructive pulmonary disease, diabetes, heart failure, ischemic heart disease, and/or chronic kidney disease. Traditional Medicare costs are total Medicare-reimbursed (ie, paid) Part A and Part B costs. ^b^Among beneficiaries with ≥1 CCW flag in 2023 for the chronic conditions identified in note “a.” E&M visits identified from these beneficiaries’ physician/professional (Part B) and outpatient (Part A) claims in 2023, using HCPCS codes defined by CMS for attributing patients to ACOs or clinicians under the Medicare Shared Savings Program, Merit-based Incentive Payment System, and other alternative payment models, including revenue codes for federally qualified health centers, rural health clinics, critical access hospitals, and community mental health centers. Primary care clinician providers defined as physician and non-physician clinicians eligible for beneficiary assignment to ACOs under the Medicare Shared Savings Program and identified as primary care for the purposes of attribution, as well as federally qualified health centers, rural health clinics, and critical access hospitals in Part A. Medical specialist providers defined as specialist clinicians eligible for beneficiary assignment to ACOs under the Medicare Shared Savings Program, as well as community mental health centers in Part A. Among beneficiaries with ≥1 E&M visit, the predominant provider was identified as the clinician (NPI) who provided the most E&M visits to the beneficiary in 2023; ties were broken by using the clinician with the last date of service. Abbreviations: ACO, accountable care organization; CCW, Medicare Chronic Conditions Data Warehouse; CMS, Centers for Medicare and Medicaid Services; E&M, evaluation and management; HCPCS, Healthcare Common Procedure Coding System; NPI, National Provider Identifier.

However, the ACO model centers around primary care providers (PCPs) and insufficiently recognizes the central role that specialists play for many beneficiaries with chronic conditions.^3^ This limits ACOs’ ability to accomplish policymakers’ goals of influencing care and spending for chronic conditions.

In this commentary, we (1) highlight how a PCP-centric ACO model does not fit the specialist-centric care patterns of many TM patients with chronic conditions, (2) explain why Medicare ACO programs must adapt to this reality, and (3) recommend updating Medicare ACO programs to assign greater accountability for beneficiaries with chronic conditions to specialists.

## A PCP-centric model does not fit care patterns for many Medicare patients

The ACO model draws from the health maintenance organization (HMO) prototype wherein PCPs serve as gatekeepers who manage patients’ care under the assumption that PCPs are best positioned to understand and address care needs. This assumption informs the beneficiary attribution methods for Medicare ACOs, which prioritize assignment of cost and quality accountability to PCPs over specialists.^4^

However, Medicare beneficiaries with major chronic conditions such as heart failure require, and benefit from, regular care from specialists such as cardiologists in addition to PCPs.^5^ Among TM beneficiaries with asthma, COPD, diabetes, heart failure, ischemic heart disease, or chronic kidney disease in 2023, 53.3% of their evaluation and management (E&M) visits were with primary care clinicians, 18.5% with chronic disease medical specialists such as cardiologists and nephrologists, and the rest with other specialists (Figure 1). From a provider accountability perspective, 65.7% of these beneficiaries had a primary care clinician as their predominant provider of E&M visits, but a sizable 15.7% had a medical subspecialist as their predominant provider.

## ACO programs must adapt to existing care patterns

Traditional Medicare beneficiaries with major chronic conditions more frequently rely on medical specialists as their predominant care provider due to their complex care needs and the lack of HMO-style gatekeeping and network rules in TM. These 2 realities are unlikely to change. Adequate disease-specific care often requires more expertise in addition to what a generalist might provide.^5^ Patients with major chronic conditions require more treatment, including specialty care. Many Medicare beneficiaries enroll in TM instead of Medicare Advantage (MA) because they do not want to experience managed-care networks and gatekeeping.

For the ACO model to improve quality and limit spending growth for chronic conditions, Medicare ACO policies must adapt to real-world care patterns. In response to challenges in engaging specialists in ACOs,^6,7^ CMS launched an array of episode- and disease-based non-ACO alternative payment models (APMs). However, few specialists in TM participate in such APMs.^7^ Instead, engaging specialists in the ACO model while redesigning the model to better align with specialists’ role in caring for beneficiaries with chronic conditions should be a focus of CMS’ accountable care strategy.

## Updating ACO programs to assign greater accountability to specialists

Primary care is crucial to patient care, but existing ACO policies often give primary care precedence over specialty care—including for beneficiaries with major chronic conditions who have specialists as their predominant care providers. It is unfair and ineffective to hold PCPs financially accountable in ACO models for the population health care costs and outcomes of high-cost patients over whom PCPs have less influence than specialists. In a recent study, we found that, although PCPs were the predominant provider of care for 19.2% of their Medicare patients with complex chronic conditions, they were potentially accountable for 29.8% of the same patients under ACO-style assignment mechanisms.^8^ The CMS’ accountable care strategy should instead reflect care patterns of beneficiaries with chronic conditions by incentivizing and assigning specialists accountability for such patients within ACOs.

To grow accountable care relationships within TM, we recommend 3 specific changes. First, for beneficiaries with chronic conditions, policymakers should eliminate the existing hierarchy that prioritizes care from PCPs over specialists in the beneficiary attribution process for ACO programs.^4^ Instead, accountability for beneficiaries with chronic conditions should be attributed to their predominant provider of care that provided the most E&M visits—whether they are a PCP or specialist. Doing so will better reflect who should be accountable for the costs and care of patients with chronic conditions. To ensure that such beneficiaries are attributed to specialists with the clinical training and expertise to manage their care on an ongoing basis, we recommend making this change only for those medical specialists who are already eligible for beneficiary attribution in ACO programs, most of whom are internal medicine subspecialists.^4^

Second, policymakers should better incentivize primary and specialty team-based care in existing ACO models. Realistically, health systems are the primary environments within which such team-based care can occur. Systems have the potential to enable better coordination and less fragmentation of care for patients with chronic conditions who inevitably see more specialists. Policymakers can incentivize this with a streamlined set of ACO quality measures focused on care transitions and coordination, such as follow-up within 7 days of hospital discharge. To accomplish these goals, both specialists and PCPs should ideally face the same set of incentives. Population-based global payment for beneficiaries under the care of specialists and PCPs via risk-based ACO contracts with the health systems to which they belong are 1 potential way to accomplish this.

Third, and finally, policymakers should phase out voluntary, episode-based, and disease-based APMs in TM that have allowed health systems to selectively place affiliated primary and specialty care practices in different payment programs subject to different incentives. Instead, CMS should discontinue such APMs and assign global accountability for the population health care costs and outcomes of beneficiaries to health systems so that primary and specialty care practices in the same system face the same set of incentives. As a transitional step, episode- and disease-based APMs could be nested within existing ACO models—for example, by incorporating episode-based targets within a total cost-of-care framework. This is a logical extension of CMS’ Transforming Episode Accountability Model (TEAM) model. This would also provide a more coherent and cost-efficient administrative strategy for operating these models.

## Conclusion

The ACO model is designed around a model of care centered on PCPs rather than specialists, limiting its ability to engage Medicare beneficiaries with chronic conditions and the specialists who care for them. To accomplish policymakers’ accountable care goals, we recommend updating ACO policies to better reflect the real-world care patterns of beneficiaries with chronic conditions and incentivize specialist engagement within ACOs.

## Supplementary Material

qxaf228_Supplementary_Data
